# *N*-[Amino(imino)methyl]uronium tetrafluoroborate

**DOI:** 10.1107/S1600536812010665

**Published:** 2012-03-17

**Authors:** Michaela Fridrichová, Jan Fábry, Karla Fejfarová, Radmila Krupková, Přemysl Vaněk

**Affiliations:** a Department of Inorganic Chemistry, Faculty of Science, Charles University in Prague, Hlavova 2030, 12843 Prague 2, Czech Republic; b Inst. of Physics of the Czech Academy of Sciences, v. v. i., Na Slovance 2, 182 21 Praha 8, Czech Republic

## Abstract

In the title compound, C_2_H_7_N_4_O^+^·BF_4_^−^, inter­molecular N—H⋯O hydrogen bonds connect the cations into chains parallel to the *c* axis, with graph-set motif *C*(4). These chains are in turn connected into a three-dimensional network by inter­molecular N—H⋯F hydrogen bonds. The B—F distances distances in the anion are not equal.

## Related literature

For the non-centrosymmetric structure, containing a 2-carbamoylguanidinium cation, that is promising for applications in non-linear optics, see: Fridrichová, Němec, Císařová & Chvostová (2010 ▶); Fridrichová, Němec, Císařová & Němec (2010 ▶); Kroupa & Fridrichová (2011 ▶). For related stuctures and a detailed description of the preparation of the title cation, see: Fábry *et al.* (2012*a* ▶,*b* ▶,*c* ▶). For structures with rather strong N—H⋯F hydrogen bonds, see: Ali *et al.* (2007 ▶); Bardaji *et al.* (2002 ▶); Blue *et al.* (2003 ▶); Byrne *et al.* (2008 ▶); Zhao & Betley (2011 ▶). For information on fluorine as acceptor in organic hydrogen bonds, see: Dunitz & Taylor (1997 ▶). For hydrogen-bond classification and graph-set motifs, see: Desiraju & Steiner (1999 ▶); Etter *et al.* (1990 ▶). For a description of the Cambridge Structural Database (CSD), see: Allen (2002 ▶). For the extinction correction, see: Becker & Coppens (1974 ▶).
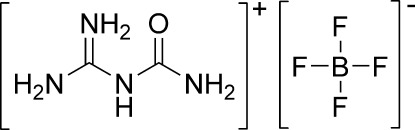


## Experimental

### 

#### Crystal data

C_2_H_7_N_4_O^+^·BF_4_^−^
*M* *_r_* = 189.9
Monoclinic, 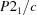

*a* = 7.8409 (3) Å
*b* = 9.6373 (4) Å
*c* = 9.5199 (4) Å
β = 105.689 (3)°
*V* = 692.57 (5) Å^3^
*Z* = 4
Cu *K*α radiation
μ = 1.86 mm^−1^
*T* = 120 K
0.51 × 0.30 × 0.17 mm

#### Data collection

Agilent Xcalibur diffractometer with an Atlas (Gemini ultra Cu) detector
Absorption correction: multi-scan (*CrysAlis PRO*; Agilent, 2010 ▶) *T*_min_ = 0.534, *T*_max_ = 0.733
7265 measured reflections
1230 independent reflections
1189 reflections with *I* > 3σ(*I*)
*R*_int_ = 0.020

#### Refinement

*R*[*F* ^2^ > 2σ(*F* ^2^)] = 0.024
*wR*(*F* ^2^) = 0.081
*S* = 1.74
1230 reflections
131 parameters
Only H-atom coordinates refined
Δρ_max_ = 0.13 e Å^−3^
Δρ_min_ = −0.15 e Å^−3^

### 

Data collection: *CrysAlis PRO* (Agilent, 2010 ▶); cell refinement: *CrysAlis PRO*; data reduction: *CrysAlis PRO*; program(s) used to solve structure: *SIR97* (Altomare *et al.*, 1997 ▶); program(s) used to refine structure: *JANA2006* (Petříček *et al.*, 2007 ▶); molecular graphics: *DIAMOND* (Brandenburg & Putz, 2005 ▶) and *PLATON* (Spek, 2009 ▶); software used to prepare material for publication: *JANA2006*.

## Supplementary Material

Crystal structure: contains datablock(s) global, I. DOI: 10.1107/S1600536812010665/lh5427sup1.cif

Structure factors: contains datablock(s) I. DOI: 10.1107/S1600536812010665/lh5427Isup2.hkl

Supplementary material file. DOI: 10.1107/S1600536812010665/lh5427Isup4.smi

Supplementary material file. DOI: 10.1107/S1600536812010665/lh5427Isup4.cml

Additional supplementary materials:  crystallographic information; 3D view; checkCIF report

## Figures and Tables

**Table 1:** Selected bond lengths (Å)

B1—F1	1.3899 (15)
B1—F2	1.3852 (12)
B1—F3	1.3754 (14)
B1—F4	1.4229 (13)

**Table 2:** Hydrogen-bond geometry (Å, °)

*D*—H⋯*A*	*D*—H	H⋯*A*	*D*⋯*A*	*D*—H⋯*A*
N1—H1*n*1⋯F4^i^	0.863 (14)	2.231 (16)	3.0069 (12)	149.5 (13)
N1—H2*n*1⋯F4^ii^	0.828 (18)	2.230 (16)	2.9666 (13)	148.5 (14)
N2—H1*n*2⋯O1^iii^	0.833 (17)	2.070 (15)	2.7981 (12)	145.7 (12)
N3—H1*n*3⋯F4^iv^	0.873 (15)	2.104 (14)	2.9286 (11)	157.4 (13)
N3—H2*n*3⋯F3^v^	0.850 (15)	2.375 (17)	2.9102 (13)	121.5 (12)
N3—H2*n*3⋯O1	0.850 (15)	2.020 (13)	2.6555 (11)	130.9 (15)
N4—H1*n*4⋯F1^iv^	0.844 (15)	2.229 (16)	3.0499 (12)	164.5 (13)
N4—H2*n*4⋯F2^iii^	0.812 (17)	2.299 (15)	2.9700 (13)	140.4 (12)
N1—H2*n*1⋯F1^vi^	0.828 (18)	2.488 (16)	2.9927 (13)	120.4 (12)
N3—H2*n*3⋯F3^v^	0.850 (15)	2.375 (17)	2.9102 (13)	121.5 (12)
N4—H2*n*4⋯O1^iii^	0.812 (17)	2.655 (15)	3.1813 (13)	123.9 (11)

Symmetry codes: (i) 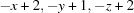; (ii) 
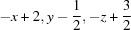
; (iii) 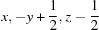; (iv) 
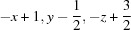
; (v) 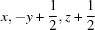; (vi) 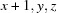.

**Fig. 1.**
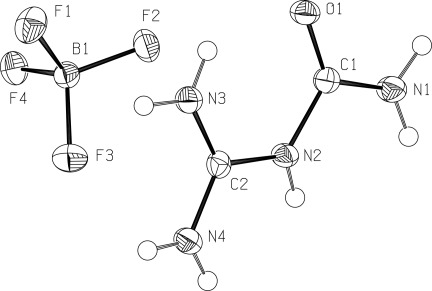
The asymmetric unit of (I), the displacement ellipsoids are depicted at the 50% probability level (Spek, 2009).

**Fig. 2.**
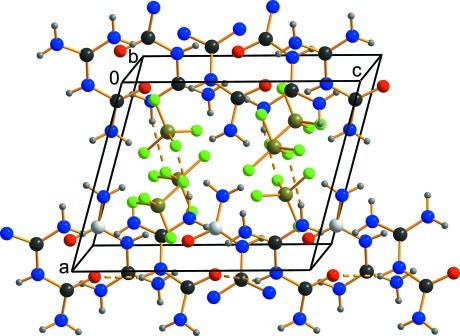
View of the unit cell of the title structure along the b axis. Colours: C (black), H (gray), B (khaki), F (green), N (blue), O (red). There are shown the strogenst hydrogen bonds in the structure N3—H1n3···F4i as well as N2—H1n2···O1ii hydrogen bonds - see Tab. 1 (Brandenburg & Putz, 2005). [Symmetry codes: i: -x + 1, y - 1/2, -z + 3/2; ii: x, -y + 1/2, z - 1/2.]

**Fig. 3.**
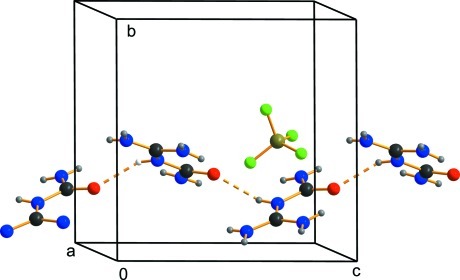
The graph set motif C(4) (Etter et al., 1990) which involves the symmetry equivalent hydrogen bonds N2—H1n2···O1ii [Symmetry code: ii: x, -y + 1/2, z - 1/2)]. The colours are the same as in Fig. 2 (Brandenburg & Putz, 2005).

## supplementary crystallographic information

### Comment

The title structure was synthesized as a part of a study of 2-carbamoylguanidinium salts with the goal of preparing non-centrosymmetric crystals which might be suitable as non-linear optical elements. 2-carbamoylguanidinium hydrogen phosphite (Fridrichová, Němec, Císařová & Chvostová, 2010; Fridrichová, Němec, Císařová & Němec, 2010; Kroupa & Fridrichová, 2011) can be given as such an example.

Another goal of this study was consideration of influence of fluorine on the hydrogen-bond pattern. Usually, especially in presence of oxygen atoms as potential acceptors, fluorine avoids involvement in hydrogen bonds in organic compounds (Dunitz & Taylor, 1997). Poor involvement in hydrogen bonds is not, however, only limited to fluorine present in organic molecules but also in inorganic molecules. Recently synthesized compounds tris(2-carbamoylguanidinium) hydrogen fluorophosphonate fluorophosphonate monohydrate (Fábry *et al.*, 2012*a*), two polymorphs of bis(2-carbamoylguanidinium) fluorophosphonate dihydrate (Fábry *et al.*, 2012*b*) and mixed crystals of 2-carbamoylguanidinium with hydrogen fluorophosphonate and hydrogen phosphite in the ratios 1:0, 0.76 (2):0.24 (2) and 0.115 (7):0.885 (7) (Fábry *et al.*, 2012*c*) exhibited weak N—H···F interactions which were weaker than the competing N—H···O hydrogen bonds in these structures.

On the other hand, inspection of the data in the Cambridge Structural Database (CSD; Version 5.32, October 2011 update; Allen, 2002) has shown that there is a number of structures which exhibit contacts N—H···F with the distances H···acceptor about 2.1 Å. It seems that majority of these structures contain highly symmetric anions with more F atoms as ligands such as [BF_4_]^-^, [PF_6_]^-1^, [SiF_6_]^2-^, [FeF_6_]^3-^*etc.* or organic molecules with a large number of fluorine substituents. ADOSIW, bis(2-ammonioethyl)ammonium hexafluoro-iron(III) monohydrate, Ali *et al.* (2007); AFUBIM, (2-aminothiazoline-*N*)-tris(pentafluorophenyl)-gold(III), Bardaji *et al.* (2002); AGOMEP, 3-(((4-methylphenyl)carbamoyl)amino)pyridinium hemikis(hexafluorosilicate, Byrne *et al.* (2008); AKEFEB, (1,2-bis(di-*t*-butylphosphino)ethane)-(aniline)-copper(I) tetrafluoroborate, Blue *et al.* (2003); ALIBED, (µ3–1,1,1-tris((2-aminoanilino)methyl)ethane)-tris(trimethylphosphino)- tri-iron hexafluorophosphate tetrahydrofuran solvate, Zhao *et al.* (2011) can be given as such examples. The reason for the involvement of F into the hydrogen-bond pattern in these structures seems to be steric: In whatever orientation fluorines get into a closer contact with the cationic H atoms.

This was also the case for the consideration of preparation of the title structure with a tetrahedral [BF_4_]^-^ anion which would enhance a chance for the formation of a stronger N—H···F hydrogen bond even in presence of the competing carbonyl group in the 2-carbamoylguanidinium cation.

Indeed, a relatively strong N3—H1*n*3···F4^i^ hydrogen bond appears in the title structure (Fig. 2) which is comparable by its geometric features to other present hydrogen bonds with the carbonyl oxygen involved, such as N2—H1*n*2···O1^ii^; N3—H2*n*3···O1; N4—H2*n*4···O1^ii^ (Table 2). [The classification of the hydrogen bonds was taken from Desiraju & Steiner (1999); the symmetry codes i: -*x* + 1, *y* - 1/2, -*z* + 3/2; ii: *x*, -*y* + 1/2, *z* - 1/2.] The valence angle B1—F4···H1N3^iii^ [the symmetry code iii: -*x* + 1, *y* + 1/2, -*z* + 3/2] equals to 109.2 (4)°. This angle would support the view that the N3—H1*n*3···F4^i^ is indeed a true hydrogen bond.

The symmetry equivalent N1—H1*n*2···O1 hydrogen bonds form chains extended along the unit-cell axis *c* (Fig. 3). The pertinent graph set motif is C(4) (Etter *et al.*, 1990). It is of interest that the secondary amine forms stronger N—H···O hydrogen bonds than the primary amines also in tris(2-carbamoylguanidinium) hydrogen fluorophosphonate fluorophosphonate monohydrate (Fábry *et al.*, 2012*a*), 2-carbamoylguanidinium with hydrogen fluorophosphonate (Fábry *et al.*, 2012*c*) and in the first polymorph of bis(2-carbamoylguanidinium) fluorophosphonate dihydrate and the first independent cation of the second polymorph of bis(2-carbamoylguanidinium) fluorophosphonate dihydrate (Fábry *et al.*, 2012*b*).

In the title structure, in addition to the hydrogen bonds with the H···acceptor distances up to ~2.2 Å there are also present interactions with the H···acceptor distances up to ~2.6 Å with low N—H···acceptor angles.

The B—F distances within the anion show considerable spread. Such a distribution of the B—F distances in the tetrafluoroborate anions is, however, usual as it was checked in the structures stored in the Cambridge Structural database (CSD; Version 5.32, October 2011 update; Allen, 2002; Tab. 1).

The χ^2^ index through the cation's non-hydrogen atoms of the title structure equals to 4671.953. This is less than in the most probably less stable polymorph of bis(2-carbamoylguanidinium) fluorophosphonate dihydrate (Fábry *et al.*, 2012*b*) with the χ^2^ index of 19477.0, and in 2-carbamoylguanidinium hydrogen phosphite with the χ^2^ index of 6515.041 (Fridrichová, Němec, Císařová & Němec, 2010; Fábry *et al.*, 2012*c*). On the other hand, it is considerably more than *e. g.* the corresponding values regarding the two independent molecules in the presumably more stable polymorph of bis(2-carbamoylguanidinium) fluorophosphonate dihydrate (Fábry *et al.*, 2012*b*) where the χ^2^ indices equal to 36.29 and 84.29.

### Experimental

The structures were prepared by neutralization of equimolar amounts of solutions of 2-carbamoylguanidinium hydroxide and tetrafluoroborate acid HBF_4_ (Sigma-Aldrich). The solutions contained about 0.89 g of 2-carbamoylguanidinium hydroxide and about 1.36 g of 48% (weight) HBF_4_.

2-carbamoylguanidinium hydroxide was prepared from 2-carbamoylguanidinium hydrochloride hemihydrate by the exchange reaction on anex (Dowex Serva, type 2X8; ion exchange OI/OH, Entwicklungslabor, Heidelberg, Germany). The preparation of 2-carbamoylguanidinium chloride hemihydrate has been described in detail in the article by Fábry *et al.* (2012*c*).

The volume of the solution after neutralization was about 30 ml. Tiny crystals floating in the solution appeared in a few days. However, they disappeared in the course of time while being replaced by a white powder. The crystal used for the structure analysis was grown from a drop of the mother liquor on a glass. (The glass was not seemingly affected by the solution.) The powder is a different compound or phase because another grown crystal dissolved in a drop that contained the particles of the powder. The obtained crystals were colourless plates with dimensions of several tenths of mm.

The calorimetric experiments were performed on differential scanning calorimeters Perkin Elmer DSC 7 (93–323 K) and PerkinElmer Pyris Diamond DSC (298–493 K). Pyris Software (Version 4.02, PerkinElmer Instruments, 2001) was used for control and evaluation. The sample (m = 11 mg) was hermetically closed in an aluminium 30µl pan, the scanning rate was 10 K/min. The DSC sample holder was purged by helium (DSC 7) or nitrogen (Pyris Diamond). Below room temperature a tiny peak is observed at 267K on heating, probably because of a residue of water. Above room temperature, a distinct exothermic peak with an endothermic onset was found at about 463K on the first heating. This exothermic reaction obviously changed the composition of the sample because two peaks that had not been observed on the very first heating were observed in subsequent runs both on heating (at 422K and 477K) and on cooling (at 409K and 462K). The first peak indicated a solid state structural phase transition while the second one could be attributed to melting or solidification.

### Refinement

All the hydrogen atoms were found in the difference electron density map and their coordinates were refined independently. The isotropic atomic displacement parameters of the hydrogen atoms were set as 1.2×*U*_eq_(N_carrier_).

### Crystal data

**Table:**

C_2_H_7_N_4_O^+^·BF_4_^−^	*F*(000) = 384
*M**_r_* = 189.9	*D*_x_ = 1.821 Mg m^−^^3^
Monoclinic, *P*2_1_/*c*	Cu *K*α radiation, λ = 1.5418 Å
Hall symbol: -P 2ybc	Cell parameters from 5746 reflections
*a* = 7.8409 (3) Å	θ = 4.6–67°
*b* = 9.6373 (4) Å	µ = 1.86 mm^−^^1^
*c* = 9.5199 (4) Å	*T* = 120 K
β = 105.689 (3)°	Irregular shape, colourless
*V* = 692.57 (5) Å^3^	0.51 × 0.30 × 0.17 mm
*Z* = 4	

### Data collection

**Table:**

Agilent Xcalibur diffractometer with an Atlas (Gemini ultra Cu) detector	1230 independent reflections
Radiation source: Enhance Ultra (Cu) X-ray Source	1189 reflections with *I* > 3σ(*I*)
Mirror monochromator	*R*_int_ = 0.020
Detector resolution: 10.3784 pixels mm^-1^	θ_max_ = 67.1°, θ_min_ = 5.9°
Rotation method data acquisition using ω scans	*h* = −9→9
Absorption correction: multi-scan (*CrysAlis PRO*; Agilent, 2010)	*k* = −11→11
*T*_min_ = 0.534, *T*_max_ = 0.733	*l* = −11→10
7265 measured reflections	

### Refinement

**Table:**

Refinement on *F*^2^	Only H-atom coordinates refined
*R*[*F*^2^ > 2σ(*F*^2^)] = 0.024	Weighting scheme based on measured s.u.'s *w* = 1/(σ^2^(*I*) + 0.0016*I*^2^)
*wR*(*F*^2^) = 0.081	(Δ/σ)_max_ = 0.006
*S* = 1.74	Δρ_max_ = 0.13 e Å^−^^3^
1230 reflections	Δρ_min_ = −0.15 e Å^−^^3^
131 parameters	Extinction correction: B-C type 1 Lorentzian isotropic (Becker & Coppens, 1974)
0 restraints	Extinction coefficient: 1600 (300)
7 constraints	

### Special details

**Table:**

Experimental. *CrysAlisPro* (Agilent Technologies, 2010) Empirical absorption correction using spherical harmonics, implemented in SCALE3 ABSPACK scaling algorithm.
Refinement. The refinement was carried out against all reflections. The conventional *R*-factor is always based on *F*. The goodness of fit as well as the weighted *R*-factor are based on *F* and *F*^2^ for refinement carried out on *F* and *F*^2^, respectively. The threshold expression is used only for calculating *R*-factors *etc*. and it is not relevant to the choice of reflections for refinement. The program used for refinement, Jana2006, uses the weighting scheme based on the experimental expectations, see _refine_ls_weighting_details, that does not force *S* to be one. Therefore the values of *S* are usually larger than the ones from the *SHELX* program.

### Fractional atomic coordinates and isotropic or equivalent isotropic displacement parameters (Å^2^)

**Table:**

	*x*	*y*	*z*	*U*_iso_*/*U*_eq_	
B1	0.66007 (15)	0.44707 (12)	0.78507 (13)	0.0190 (4)	
F1	0.54351 (8)	0.40448 (7)	0.86358 (7)	0.0295 (3)	
F2	0.83067 (8)	0.45670 (7)	0.87576 (7)	0.0254 (2)	
F3	0.65411 (9)	0.35814 (7)	0.67085 (7)	0.0304 (3)	
F4	0.60448 (8)	0.58120 (6)	0.72842 (7)	0.0242 (2)	
N1	1.29213 (13)	0.25144 (10)	0.99490 (11)	0.0230 (3)	
H1n1	1.3573 (19)	0.2796 (14)	1.0783 (17)	0.0276*	
H2n1	1.3380 (19)	0.2346 (15)	0.9280 (17)	0.0276*	
C1	1.12706 (13)	0.21215 (10)	0.98698 (11)	0.0180 (3)	
O1	1.05904 (10)	0.21991 (8)	1.08889 (8)	0.0212 (3)	
N2	1.03592 (11)	0.16014 (9)	0.85077 (9)	0.0183 (3)	
H1n2	1.0786 (18)	0.1719 (14)	0.7805 (17)	0.022*	
C2	0.86829 (13)	0.10769 (10)	0.81483 (11)	0.0179 (3)	
N3	0.77643 (12)	0.10138 (10)	0.91111 (10)	0.0211 (3)	
H1n3	0.666 (2)	0.0746 (14)	0.8846 (15)	0.0253*	
H2n3	0.8179 (18)	0.1386 (15)	0.9944 (17)	0.0253*	
N4	0.80477 (13)	0.06150 (10)	0.68075 (11)	0.0221 (3)	
H1n4	0.702 (2)	0.0267 (15)	0.6529 (15)	0.0265*	
H2n4	0.8632 (19)	0.0642 (14)	0.6222 (16)	0.0265*	

### Atomic displacement parameters (Å^2^)

**Table:**

	*U*^11^	*U*^22^	*U*^33^	*U*^12^	*U*^13^	*U*^23^
B1	0.0181 (6)	0.0208 (6)	0.0181 (6)	0.0008 (4)	0.0052 (5)	0.0009 (4)
F1	0.0255 (4)	0.0333 (4)	0.0341 (4)	0.0011 (3)	0.0155 (3)	0.0082 (3)
F2	0.0189 (4)	0.0318 (4)	0.0229 (4)	0.0015 (2)	0.0015 (3)	0.0031 (2)
F3	0.0343 (4)	0.0309 (4)	0.0255 (4)	0.0008 (3)	0.0074 (3)	−0.0079 (3)
F4	0.0228 (4)	0.0227 (4)	0.0254 (4)	0.0021 (2)	0.0033 (3)	0.0046 (2)
N1	0.0199 (5)	0.0296 (5)	0.0203 (5)	−0.0050 (4)	0.0070 (4)	−0.0057 (4)
C1	0.0203 (5)	0.0156 (5)	0.0177 (5)	0.0015 (4)	0.0048 (4)	0.0008 (3)
O1	0.0222 (4)	0.0257 (4)	0.0165 (4)	−0.0024 (3)	0.0065 (3)	−0.0025 (3)
N2	0.0188 (5)	0.0231 (5)	0.0141 (5)	−0.0012 (3)	0.0060 (4)	0.0003 (3)
C2	0.0181 (5)	0.0162 (5)	0.0183 (5)	0.0030 (4)	0.0031 (4)	0.0021 (4)
N3	0.0174 (5)	0.0262 (5)	0.0196 (5)	−0.0025 (3)	0.0052 (4)	−0.0031 (4)
N4	0.0202 (5)	0.0273 (5)	0.0178 (5)	−0.0020 (4)	0.0038 (4)	−0.0029 (4)

### Geometric parameters (Å, º)

**Table:**

B1—F1	1.3899 (15)	C2—N4	1.3159 (14)
B1—F2	1.3852 (12)	N1—H1n1	0.863 (14)
B1—F3	1.3754 (14)	N1—H2n1	0.828 (18)
B1—F4	1.4229 (13)	N2—H1n2	0.833 (17)
N1—C1	1.3309 (15)	N3—H1n3	0.873 (15)
C1—O1	1.2293 (14)	N3—H2n3	0.850 (15)
C1—N2	1.3936 (12)	N4—H1n4	0.844 (15)
N2—C2	1.3627 (13)	N4—H2n4	0.812 (17)
C2—N3	1.3113 (16)		
			
F1—B1—F2	110.44 (9)	N3—C2—N4	121.80 (10)
F1—B1—F3	110.76 (9)	H1n1—N1—H2n1	119.8 (15)
F1—B1—F4	107.07 (9)	H1n3—N3—H2n3	119.6 (15)
F2—B1—F3	110.80 (9)	H1n4—N4—H2n4	117.5 (14)
F2—B1—F4	108.70 (8)	H1n1—N1—C1	117.8 (11)
F3—B1—F4	108.97 (9)	H2n1—N1—C1	121.3 (9)
N1—C1—O1	124.11 (9)	C1—N2—H1n2	118.8 (9)
N1—C1—N2	113.66 (10)	H1n2—N2—C2	114.7 (9)
O1—C1—N2	122.24 (9)	C2—N3—H1n3	120.0 (10)
C1—N2—C2	125.74 (10)	C2—N3—H2n3	119.2 (11)
N2—C2—N3	121.10 (9)	C2—N4—H1n4	121.2 (11)
N2—C2—N4	117.09 (11)	C2—N4—H2n4	121.3 (9)

### Hydrogen-bond geometry (Å, º)

**Table:**

*D*—H···*A*	*D*—H	H···*A*	*D*···*A*	*D*—H···*A*
N1—H1*n*1···F4^i^	0.863 (14)	2.231 (16)	3.0069 (12)	149.5 (13)
N1—H2*n*1···F4^ii^	0.828 (18)	2.230 (16)	2.9666 (13)	148.5 (14)
N2—H1*n*2···O1^iii^	0.833 (17)	2.070 (15)	2.7981 (12)	145.7 (12)
N3—H1*n*3···F4^iv^	0.873 (15)	2.104 (14)	2.9286 (11)	157.4 (13)
N3—H2*n*3···F3^v^	0.850 (15)	2.375 (17)	2.9102 (13)	121.5 (12)
N3—H2*n*3···O1	0.850 (15)	2.020 (13)	2.6555 (11)	130.9 (15)
N4—H1*n*4···F1^iv^	0.844 (15)	2.229 (16)	3.0499 (12)	164.5 (13)
N4—H2*n*4···F2^iii^	0.812 (17)	2.299 (15)	2.9700 (13)	140.4 (12)
N1—H2*n*1···F1^vi^	0.828 (18)	2.488 (16)	2.9927 (13)	120.4 (12)
N3—H2*n*3···F3^v^	0.850 (15)	2.375 (17)	2.9102 (13)	121.5 (12)
N4—H2*n*4···O1^iii^	0.812 (17)	2.655 (15)	3.1813 (13)	123.9 (11)

Symmetry codes: (i) −*x*+2, −*y*+1, −*z*+2; (ii) −*x*+2, *y*−1/2, −*z*+3/2; (iii) *x*, −*y*+1/2, *z*−1/2; (iv) −*x*+1, *y*−1/2, −*z*+3/2; (v) *x*, −*y*+1/2, *z*+1/2; (vi) *x*+1, *y*, *z*.
